# Production of alkaline lipase by *Aspergillus terreus* AUMC 15762 for laundry application

**DOI:** 10.1186/s13568-025-01865-x

**Published:** 2025-04-13

**Authors:** Osama Abdel-Hafeez Mohamed Al-Bedak, Ahmed Mohamed Ahmed Ali Ramadan, Hussein H. EL-Sheikh, Reda M. Shehata

**Affiliations:** 1 Assiut University Mycological Centre, Assiut, 71511 Egypt; 2 ERU Science and Innovation Center of Excellence, Egyptian Russian University, Badr City, 11829 Egypt; 3 Department of Botany and Microbiology, Faculty of Science, Al Azhar University, Cairo, 11511 Egypt

**Keywords:** *Aspergillus*, Laundry, Lipase, Optimization, Plackett–Burman, Purification, Submerged

## Abstract

Lipases are extraordinarily critical co-factor-independent enzymes with profound economic consequences. They are utilized extensively in production of fine chemicals, food, textile, pulp and paper, laundry, and biodiesel sectors. In the current study, the lipolytic activity of 141 fungal isolates—representing 21 genera and 38 species—that were isolated from samples of desert soil gathered from the Governorates of Sohag, Qena, and Aswan were examined. Of the 74 isolates showed positive lipase activity, 40 were high lipase producers. In terms of lipase production, *Aspergillus terreus* AUMC 15762 was the most effective strain. To enhance the synthesis of lipase from *Aspergillus terreus* AUMC 15762, Plackett–Burman design (PBD) was employed. For the maximal amount of lipase synthesis (103.3 U/mL), ammonium sulphate was required after three days at 25 °C, pH 4.0, and 3.0 g/L. Through the use of Trilite MA 12 anion exchanger and Sephadex G-100 column chromatography, lipase was purified 17.79 times and achieved 64.714 kDa molecular weight on SDS-PAGE. The highest possible specific activity of 3867.85 ± 214.28 U/mg was attained at pH 8.0 and 40 °C. The addition of KCl and ZnSO_4_ raised the lipase specific activity by 115.42%. K_m_ of 19.0 mg/mL and V_max_ of 1000 μmol/min were determined for the pure lipase. The effects of 20 U/mL pure lipase on corn and olive oily spots were examined in this work at pH 8.0 and 40 °C. The pure lipase completely removed oil contamination from fiber surfaces, as evidenced by the oily spots’ separation from the white cotton textiles after 60 min. This work offers a lipase produced from *Aspergillus terreus* species that showed promise for industrial laundry applications.

**Supplementary Information:**

The online version contains supplementary material available at 10.1186/s13568-025-01865-x.

## Introduction

Lipases (EC: 3.1.1.3) are an important class of enzymes for biotechnology because they may catalyze the production and degradation of triacylglycerol ester bonds (Corzo and Revah 1999). They are the third-largest group in terms of total sales volume, trailing only proteases and carbohydrolases (Kumar et al. 2023). The commercial application of lipases is worth billions of dollars. Detergents, biodiesel, dairy, fat and oil hydrolysis, organic syntheses, fat modification, flavor enhancement in food preparation, racemic mixture resolution, pharmaceutical industry, detergent additives, biofuel manufacturing, chemical analysis, and the synthesis of fine chemicals, agrochemicals, and new polymeric materials are just a few of the industries that use lipases (Kumar et al. 2023; Enespa et al. 2022; Mhetras et al. 2021).

When it comes to total sales, lipases are one of the largest types of enzymes (Fatima et al. 2021). The global market for microbial lipase was estimated to be worth USD 349.8 million in 2019. From 2020 to 2025, it is anticipated to rise at a compound yearly growth rate (CAGR) of 5.2%, reaching USD 797.7 million (Fatima et al. 2021), which makes the microbial lipase market one of the most dynamic industry sectors. The majority of the enzymes used in modern industrial processes come from microbes (Ali et al. 2023).

Among microbiological sources, filamentous fungi are good lipase producers, and the extraction, purification, and processing procedures are not overly difficult. Filamentous fungi are utilized in industrial settings because they produce extracellular lipases, which are easier to recover from the production medium and require less money to create than bacterial lipases (Martorell et al. 2019; Duarte et al. 2018; Contesini et al. 2017). Enzymes from fungi are cheaper to purchase than those from bacteria because they can be easily isolated (Mehta et al. 2017). Lipase-producing fungi that are important for trade include species of *Alternaria dianthicola* (Kakde and Chavan 2010), *Aspergillus* sp. (Hind et al. 2024), *Colletotrichum gloeosporioides* (Balaji and Ebenezer 2008), *Fusarium* sp. (Iftikhar et al. 2012), *Hypocrea pseudokoningii* (Pereira et al. 2014), *Macrophomina phaseolina* (Schinke and Germani 2012), *Mucor hiemalis* (Hiol et al. 1999), *Penicillium* sp. (Ferreira et al. 2017), and *Rhizopus* sp. (Idrees and Rajoka 2002).

Submerged fermentation in batch and fed-batch cultures is the main process used to generate industrial enzymes (Haack et al. 2006). Compared to solid-state operations, submerged fermentations offer more control over parameters like pH and temperature and a more homogenous culture medium (Pandey et al. 2000). Lipases have been made by submerged fermentation because centrifugation or simple filtration make it easier to extract extracellular enzymes and determine biomass (Coradi et al. 2013).

Because lipases are used in so many different industrial applications, it is important to study their properties because different lipases from different sources can have different properties (Maldonado 2006). An enzyme’s maximum activity depends on the integrity of its structure, so temperature, pH, chemical agents, autolysis, and ionic strength can all affect its maximum activity. Temperature also affects the kinetic energy of substrates and enzyme molecules, increasing the number of productive collisions per unit of time (Iyer and Ananthanarayan 2008).

The breakdown or denaturation of the enzyme’s tertiary structure as a result of modifications to bonds, such as hydrogen bonds, disulfide bonds, and hydrophobic interactions, can lead to the inactivation of enzymatic activity (Gomes et al. 2006). The ionization state of amino acids, which determines the secondary and tertiary structures of proteins and, consequently, their activity and stability, is altered by changes in pH, which has an impact on the stability of enzymes by altering the electrostatic contacts of their protein structure (Rajakumara et al. 2008; Sharma et al. 2002). The purpose of this study is to screen and maximize the synthesis of lipase from *Aspergillus terreus* AUMC 15762, a wild isolate from the desert soil of Upper Egypt. This was followed by the isolation and characterization of *Aspergillus terreus* AUMC 15762 lipase.

## Materials and methods

### Screening of lipolytic activity

The lipolytic activity for 141 isolates were tested using the lipase production medium suggested by Ullman and Blasins (1974). Tween 80 was autoclaved separately and added to the sterile and cooled basal medium prior to solidification. The medium was aseptically dispensed into 20 mL test tubes (12 mL each). The tubes were individually inoculated with 50 μL spore suspension obtained from a 7-day-old cultures of the tested strains. The tubes were then incubated at 28 °C for 10 days. The lipolytic ability was observed as a visible precipitate due to the formation of the calcium oleate crystals liberated as a result of the enzyme action. The depth of each visible precipitate (in mm) was measured, and the tested isolates were categorized as high (H), moderate (M), and low (L) producers.

### Morphological and molecular identification of the potent isolate

On Czapek’s Dox agar (CzA), malt extract agar (MEA), and Czapek yeast autolysate agar (CYA) (Smith and Onions 1994), *Aspergillus* strain was inoculated in three point pattern. Following seven days of incubation at 25 °C, microscopic features on MEA were examined with lactophenol cotton blue, and the strain employed in this investigation was identified based on both their macroscopic and microscopic characteristics (Raper and Fennell 1965). The strain of *Aspergillus* was kept alive in the culture collection of the Assiut University Mycological Centre as AUMC 15762.

Fungal DNA was isolated following the method outlined by Moubasher et al. (2019), and the PCR reaction was performed using SolGent EF-Taq (Al-Bedak and Moubasher 2020). ITS1 and ITS4 universal primers were used for amplification of the ITS region (White et al. 1990). DNASTAR (version 5.05) was utilized to generate consecutive sequences of the *Aspergillus* species in this investigation. The most comparable species’ sequences from the *Aspergillus* genus and placed in the section Terri were downloaded from GenBank. MAFFT (Katoh and Standley 2013) was used to align all of the sequences, and BMGE (Criscuolo and Gribaldo 2010) was used to optimize the alignment gaps and weak, uninformative characters. Phylogenetic analyses using maximum-likelihood (ML) and maximum-parsimony (MP) methods were performed using MEGA X (version 10.2.6) (Kumar et al. 2018), and the robustness of the most parsimonious trees was evaluated by 1000 replications (Felsenstein 1985). Utilizing Modeltest 3.7’s Akaike Information Criterion (AIC), the optimum nucleotide substitution model for ML analysis was identified (Posada and Crandall 1998).

### Plackett–Burman design (PBD)

With the PBD (Plackett and Burman 1946), parameters were tested at two different levels (high and low), and the major influences were identified by using statistical methods such as ANOVA to analyze the findings. A total of nine variables comprising six nutritional components (A = temperature, B = pH, C = Tween 80, D = peptone, E = yeast extract, F = sodium nitrate, G = ammonium chloride, H = urea, and J = ammonium sulphate) were evaluated at two levels (Table S1).

The primary impact of every component was computed by comparing the lipase production means at + 1 and − 1 concentrations. Based on the nutritional parameters impacting lipase production, the variables and their values were selected (Patil et al. 2021; Kazeem et al. 2024). Equation (1) displays the PBD based on the first order polynomial model.

1 $$ {\text{Y}} = \, \beta_{0} + \, \Sigma \, \beta_{{\text{i}}} {\text{X}}_{{\text{i}}} $$

where Y = the response (Lipase production in g/L), β_0_ = the model intercept, β_i_ = the linear coefficient and, X_i_ = the concentration of independent factors (i = 1, 2, 3,…, 11).

At the 95% (*p* < 0.05) confidence level for each component, the value of the coefficient βi, whether positive or negative, indicated the effect of the relevant factors on lipase synthesis. Using ANOVA, the model's significance was determined. Response surface methodology (RSM) was used to choose the elements for optimization based on which the Pareto chart indicated the greatest impact.

### Optimization of lipase production using RSM

In this experiment, three independent variables (pH, ammonium chloride and ammonium sulphate), that were selected as significant from the PBD, were optimized using Box–Behnken (BBD) of response surface methodology (RSM). Their effect on lipase production was studied and variables were tested at 3 code levels (− 1, 0, and + 1). Details of experimental plans were shown (Table S2). Using the design expert software, 29 runs of experiment were generated with five center points. Point prediction techniques were employed to optimize each factor for maximal lipase production. The model underwent iterative procedures, system identification, and parameter estimation for enhanced accuracy and reliable simulation outcomes. A second order polynomial equation generated was shown in Eq. (2).

2 $$ {\text{Y }} = \, \beta_{{\text{o}}} + \, \Sigma \, \beta_{{\text{i}}} {\text{X}}_{{\text{i}}} + \, \Sigma \, \beta_{{{\text{ij}}}} {\text{X}}_{{\text{i}}} {\text{X}}_{{\text{j}}} + \, \Sigma \, \beta_{{{\text{ii}}}} {\text{X}}_{{\text{i}}}^{{2}} $$

where Y = the predicted response, β_0_ = the model constant; X_i_ = independent factors (pH, ammonium chloride and ammonium sulphate); β_i_ and β_ii_ = coefficients. In order to determine the significance of the model terms, the data obtained from the (BBD) were subjected to ANOVA analysis.

### Lipase assay and total protein estimation

Lipase activity was determined by alkali titration using Tween 80 as substrate (Akhter et al. 2022). One mL of the culture supernatant was added to the reaction mixture that contained 3 mL of 100 mmol phosphate buffer (pH 8.0) and 3 mL of Tween 80, and the reaction mixture was incubated at 37 °C for 30 min. Both test (in which all the reaction mixture were added with enzyme) and blank (in which all the reaction mixture were added without enzyme) were performed. After 30 min, 3 mL of 95% ethanol was added to stop the reaction. The reaction mixture was then transferred to a 50-mL Erlenmeyer flask, and the liberated fatty acids were titrated against 50 mmol NaOH solution using phenolphthalein as an indicator. End point was an appearance of a light pink color. One unit of lipase activity was defined as the amount of enzyme required to release 1 µmol of free fatty acids per mL under the standard assay conditions. The lipase activity was calculated according to the Eq. (3).

3 $$ {\text{Lipase activity}} = \frac{{{\text{V}}1{ } \times {\text{ M }} \times { }1000}}{{{\text{V}}2{ } \times {\text{ T}}}} $$

where V1 = Volume NaOH consumed for the sample—volume of NaOH for the blank; M = Molarity of the NaOH titrant used (0.05 in this case); 1000  =  Conversion factor from milli-equivalent to micro-equivalent; T = time of the reaction (min); V2 = Volume of enzyme used (mL).

The concentration of proteins was determined according to the method mentioned by Lowry’s method (Lowry et al. 1951; Waterborg 2009). Bovine serum albumin standard curve was used to estimate the protein concentrations.

## Purification of lipase

### Ammonium sulfate precipitation

After the incubation time, cell-free supernatant was recovered by centrifuging at 10,000 rpm for 10 min. Using 70% saturated ammonium sulphate, total protein was extracted at 4 °C. The precipitated protein was lyophilized using a freeze dryer (VirTis, model #6KBTES-55, NY, USA). Lyophilized protein was dissolved in citrate buffer (pH 8.0), dialyzed twice for two hours at room temperature (cutoffs: 12–14 KD), and then cooled overnight at 4 °C to remove salts and other small molecules. The dialyzed protein's fungal lipase, which had been partially purified and lyophilized, was subsequently used in enzyme purification procedures.

### Ion–exchange chromatography

An anion exchanger Trilite MA-12 was positioned within a glass column (50 cm × 2.5 cm). After equilibrating the column with phosphate buffer (100 mM, pH 8.0), the enzyme sample was injected. The enzyme was then eluted at 0, 0.1, 0.25, 0.5, 1.0, and 1.5 M concentrations of NaCl using 100 mM phosphate buffer (pH 8.0). The fractions had a capacity of 6.0 mL, and the column flow rate was set at 0.25 mL/min. The activity of lipase was determined using the previously described method. The fractions that were most lipase-active were mixed, concentrated, and used for additional purification procedures.

### Sephadex G 100 gel filtration column

Sephadex G 100 gel was packed in a glass column (50 cm × 2.5 cm). After the concentrated enzyme sample was injected onto this column, the protein was eluted using phosphate buffer (100 mM, pH 8.0). The methods previously mentioned were used to assess the lipase's activity in fractions with a volume of 6.0 ml. Pooling, condensing, and lyophilizing the most active fractions was done.

### Determination of molecular weight of the pure lipase

Prior to SDS-PAGE, the recently described one-step acetone precipitation was carried out (Niu et al. 2018). A 0.01 g of the lipase powder was dissolved in 1.0 mL of Tris HCl buffer (pH 6.8), and five volumes of cold acetone (− 20 °C) were used to precipitate the protein extracts, which were then stored at − 20 °C for two pellet-washing procedures. Lipase pellets were obtained by centrifuging at 15,000 rpm for 5 min at 4 °C, and air-drying for 5 min. Afterwards, a 50 µg of the lipase sample were dissolved in 300 µL of RIPA lysis buffer, and briefly sonicated for 3 min at 60 Hz (Ultrasonic Cleaner Merk Power Sonic 405, Falc; Italy). After estimating the protein concentrations using the Bradford assay (Bradford 1976), aliquots of the protein samples were combined with sample buffer and boiled for 5 min. In addition to 5 µL of pre-stained protein marker, 30 µL of the sample (nearly 5 µg of proteins), were added to the wells in 4% stacking gel and 10% SDS (Laemmli 1970), and the running process was performed at 120 v for 60 min. Gel was removed after electrophoresis was completed, dyed with Coomassie Brilliant Blue R-250.

### Estimation of the apparent molecular weight of the pure lipase

The *Rf* value for each standard prestained marker and the target proteins was determined using ImagJ software following the gel’s scanning. The corresponding molecular weight log was plotted against the *Rf* values of the marker. The target proteins was determined by calculating the anti-log of the matching log of the target protein's *Rf* value (Matsumoto et al. 2019).

### Impact of pH, temperature and metal ions and inhibitors on the pure lipase activity

The effects of pH (3.0–11.0) at 25–60 °C (in increments of 5 ºC) on pure lipase activity have been investigated. A 0.01 g of enzyme powder (dissolved in 1.0 mL of buffer solution with the appropriate pH values) was mixed with 3.0 mL of 1.0% Tween 80 at the tested temperatures to initiate the reaction. The reaction was stopped after 30 min by adding 3.0 mL of 96% ethyl alcohol, and the contents of the flask were titrated against a 50 mmol NaOH solution. Additionally, metal ions such as Na^+^, K^+^, Ca^2+^, Mg^2+^, Mn^2+^, Fe^2+^, Cu^2+^, Zn^2+^, Ni^2+^, and Co^2+^, and were tested by adding them to a solution at a concentration of 5 mM as NaCl, KCl, CaCl_2_, MgSO_4_, MnSO_4_, FeSO_4_, CuSO_4_, ZnSO_4_, NiCl_2_, and CoCl_2_. Moreover, an enzyme inhibitor was examined using 5 mmol of ethylenediaminetetraacetic acid (EDTA) and sodium dodecyl sulphate (SDS). To ascertain what 100% activity means, the activity of the pure lipase was measured under conventional reaction conditions, without the presence of metal ions, EDTA, or SDS. Three runs of the experiment were conducted.

### Substrate specificity

Olive oil, sesame oil, sunflower oil, Tween 20, and Tween 80, each at 1% concentration, were used to measure the activity of the pure lipase produced in this study. As mentioned earlier, the lipase activity was estimated.

### Determination of K_m_ and V_max_

K_m_ (Michaelis–Menten constant) and V_max_ (maximum reaction velocity) values of the pure lipase were determined by measuring enzyme activity at different concentrations of Tween 80 (1–20 mg/mL), using the Line-weaver–Burk plot and Hofstee and Hanes–Woof plots (Michaelis and Menten 1913; Lineweaver and Burk 1934) using the Eq. (4).

4 $$\frac{1}{\text{v}}=\frac{1}{{V}_{max}}+\frac{{\text{K}}_{\text{m}}}{{V}_{max}} \times \frac{1}{\text{S}}$$

### Application of the pure lipase in removal of oily waste from clothes

Spots of maize and olive oil containing 0.1% coffee were created on clean, uncontaminated segments (10 cm × 10 cm) of white cotton cloth using the technique described by Das et al. (2016). The cotton cloth with the oily patches was left for 15 min, and then it was dried for 5 min at 80 ºC in a hot air oven. The dried spots were individually put into Erlenmeyer conical flasks that were being shaken at 150 rpm and held 20 U/mL of pure lipase in phosphate buffer (pH 8.0), which was incubated at 40 °C for 60 min. The investigated region was left to air dry after the incubation period and then rinsed with tap water for two minutes without scrubbing. The identical procedure was followed by the lipase-free control.

### Statistical analysis

The mean and standard deviation (SD) of the tentative study performed in triplicate were used to express all data. Analysis of the statistical significance was conducted according to Stahle and Wold (1989). It was deemed significant at *p* ≤ 0.05.

## Results

### Qualitative and quantitative screening of lipolytic activity

One hundred forty-one fungal isolates were examined in this test, according to the preliminary lipolytic screening results, around half of the tested fungi exhibited lipolytic activity, which expressed in the test tubes as a calcium oleate precipitate. Seventy-four of them demonstrated lipase activity. The isolates that produced lipase were divided as low (13 isolates), moderate (21 isolates), and high (40 isolates) producers. A total of 84 of the isolates were associated with *Aspergillus*, and 56 of them showed lipolytic activity, making up 59.47% of the total *Aspergillus* and 75.6% of the positive isolates overall (Table S1). For the quantitative screening of lipolytic activity, forty fungal isolates that showed highest lipase activity were selected for the assay.

In this test, a total of 141 fungal isolates were investigated. Based on the first results of the lipolytic screening, around 50% of the fungi under investigation (74 isolates) demonstrated lipolytic activity, manifesting itself as a precipitate of calcium oleate in the test tubes. There were three categories of lipase-producing isolates: low (13 isolates), moderate (21 isolates), and high (40 isolates). *Aspergillus* was linked to 56 of those isolates exhibited lipolytic activity accounting for 75.6% of the positive isolates overall and 59.47% of the total *Aspergillus*. Forty isolates with the greatest lipase activity were chosen for the assay in order to perform a quantitative screening of lipolytic activity. These forty isolates were divided into three categories based on the findings of the secondary screening: low (8 isolates), moderate (24) and high (8) producers (Table S1). The high lipase producers were *Aspergillus terreus* (3 isolates), *A. niger*, *A. tubingensis*, *A. pseudodeflectus*, *Penicillium chrysogenum*, and *P. crustosum* (one isolate each).

### Morphological and molecular identification of the potent isolate

The powerful fungus's morphological identification showed that it shared all the traits of *A. terreus*, which generated colonies with colors ranging from orange-brown to cinnamon and long, compact, columnar conidial heads carried on short, biseriate conidiophores (Fig. S1).

**Fig. 1:**
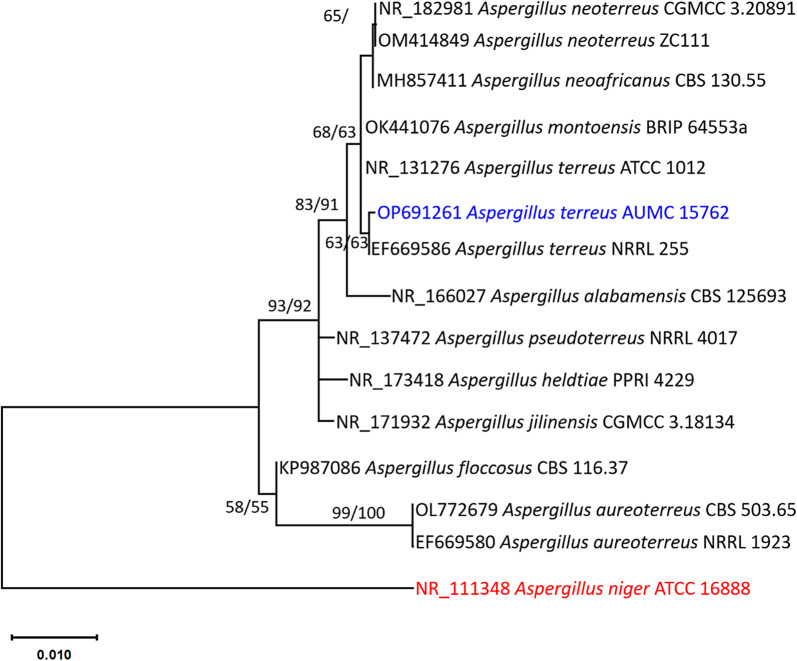
Maximum likelihood phylogenetic tree obtained from ML/MP analysis of ITS sequences of *Aspergillus terreus* AUMC 15762 in this study (in blue) compared to the most similar species of *Aspergillus terreus* group in GenBank. Bootstraps (1000 replications) for ML/MP ≥ 50% are indicated near the respective nodes. The tree is rooted to *Aspergillus niger* ATCC 16888 (in red)

ITS sequencing-based phylogenetic analysis was used. Fifteen sequences yielded a total of 605 characters in the final ITS data set, of which 512 could be accurately aligned, 57 were classified as variable, and 16 were classified as informative. To depict the link between taxa, Tamura’s 3-parameter model (T92) proved ideal. Ten trees were obtained using the Maximum Parsimony approach. The most parsimonious tree (Fig. 1) has the following characteristics: tree length of 70, highest log likelihood of −1221.77, consistency index of 0.916667, retention index of 0.954545, and composite index of 0.875000. The strain in this study located at the same branch as *A. terreus* NRRL 255 (Fig. 1). Therefore, it is identified here as *A. terreus* and sequence of its respective ITS was uploaded to GenBank as OP691261 (Fig. 1).

### Plackett–Burman design (PBD)

**Fig. 2:**
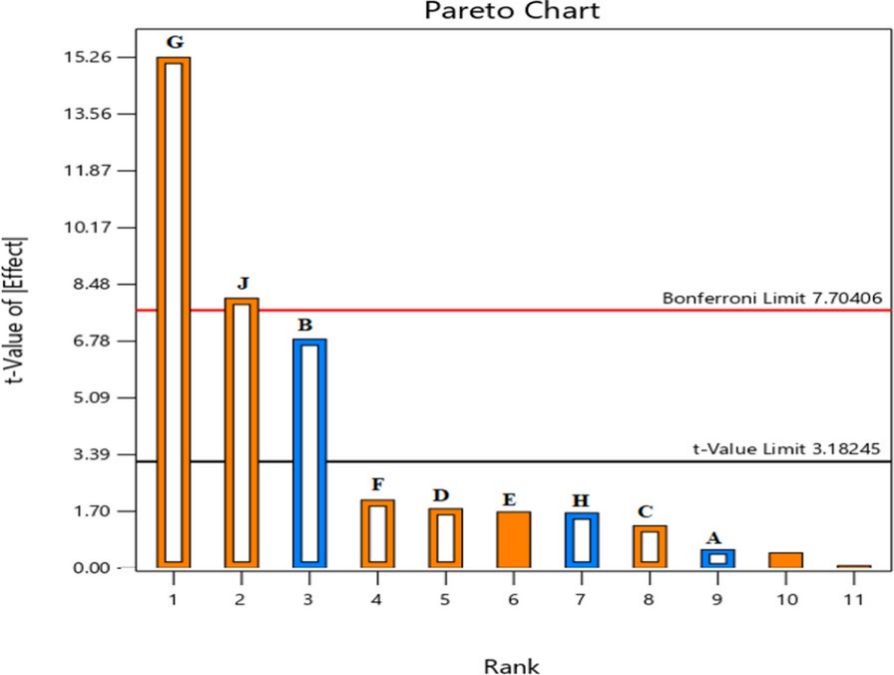
Pareto chart of the standardized effects of screened factors on Lipase activity using PBD (A = temperature; B = pH; C = Tween 80; D = peptone; F = sodium nitrate; G = ammonium chloride; H = urea; J = ammonium sulphate)

To narrow down the variables that affect lipase synthesis, the PB design was used (Table S4). The factor's effect value revealed how much of an impact it had on the response factor. To identify significant variables, a Pareto chart (Fig. 2) was created using the findings of the (PBD) screening. When the level of the tested factors was high or low, respectively, the positive or negative sign of each effect value indicated that each tested parameter influenced to get the greater production of lipase. The synthesis of lipase was negatively impacted by the factors A (temperature), B (pH), and H (urea). On the other hand, the factors C (Tween 80), D (peptone), F (sodium nitrate), G (ammonium chloride), and J (ammonium sulphate) had beneficial impacts (Fig. 2).

However, the model appears to be significant based on the F-value of 44.54. The likelihood of an F-value this large occurring owing to noise was only 0.49%. Model terms were considered significant if their *P*-value was less than 0.05. B, G, and J were important model terms in this instance. Ammonium sulphate, pH, and ammonium chloride were the three main influential factors found based on the Pareto chart. As a result, these variables were chosen for the RSM experiment in order to determine their ideal concentrations and the best settings for lipase production. The important model equation in terms of coded factors was according to Eq. (5).

5 $$ \begin{aligned} {\text{Y}} \;=\; & {3}0.{83}{-}0.{3}0{\text{62A}}{-}{3}.{\text{85 B}} + 0.{\text{7121C}} \\ & + 0.{\text{996D}} + {1}.{\text{14 F}} + {8}.{\text{59 G}} \\ & {-}0.{\text{9246 H}} + {4}.{\text{54 J}} \\ \end{aligned} $$

In this case, we eliminated non-significant variables that made it more difficult to create a meaningful mathematical model that accurately reflected the experimental data. Response predictions for predetermined levels of each factor were made possible by the coded equation. This coding makes it easier to compare factor coefficients and determine how much of an impact each has. The coefficient of determination (R^2^) was used to assess how well the model fit the data. This figure indicates the percentage of the actual response data variation that can be accounted for by the interaction of the experimental factors. A perfect fit, where the model accounts for all variation in the observed data, is represented by an R^2^ of 1. There were less than 0.2 discrepancies between the Adjusted R^2^ of 0.9694 and the Predicted R^2^ of 0.8664, indicating a reasonable agreement.

### Optimization of significant factors using bbd for maximize lipase production

Three variables that were found to have a significant impact on lipase synthesis from the PBD experiment were chosen: pH, NH_4_Cl, and (NH_4_)_2_SO_4_. Their ideal concentration and the way the factors interacted were found using the BBD. There were 17 trial runs produced. Run 14 produced the highest lipase activity (103.3 U/mL), while Run 15 produced the lowest (33.3 U/mL) (Table 1).

**Table 1:** The BBD for optimization of Lipase production

Run	pH	NH_4_Cl	(NH_4_)_2_SO_4_	Lipase (U/mL)
1	7	2	2	45.3
2	7	1	1	36.6
3	7	3	1	50.0
4	7	2	2	55.0
5	10	2	1	41.1
6	7	3	3	60.5
7	7	1	3	47.8
8	4	1	2	102.2
9	7	2	2	53.3
10	7	2	2	41.6
11	7	2	2	50.5
12	4	2	1	66.6
13	10	2	3	33.9
14	4	2	3	103.3
15	10	1	2	33.3
16	10	3	2	48.9
17	4	3	2	100

### Final equation in terms of coded factors

It is possible to predict the reaction for specific levels of each element by using the equation expressed in terms of coded factors. The factors are coded as follows by default: (+ 1) for high levels and (− 1) for low levels. By comparing the factor coefficients, the coded equation can be used to determine the relative impact of the components. Equation (6) presents the regression equation of the BBD design for Lipase production in Coded Units, which was created through the use of design expert software.

6 $$ \begin{aligned} Y\; = \; & 49.15 - 26.88{\text{A}} + 4.94{\text{B}} \\ & + 6.39{\text{C}} - 10.97{\text{AC}} + 17.22{\text{A}}^{2} \\ & + 4.72{\text{B}}^{2} - 5.14{\text{C}}^{2} \\ \end{aligned} $$

where Y is the lipase activity (U/mL) produced as a function of the coded levels of pH level (A), NH_4_Cl concentration (B), and (NH_4_)_2_SO_4_ (C), respectively.

ANOVA was used to evaluate the model's statistical significance. According to the *F*-value (34.19) and *p*-value (0.0001), the model used for the generation of lipase was statistically significant (*p* < 0.01). The coefficients (A, B, C, AC, and A^2^) showed significant *p*-values, indicating that these variables had an impact on the total output of lipase. *F*-values of the individual variables (main effects) showed that the most significant effects on lipase production were caused by pH and (NH_4_)_2_SO_4_, whereas NH_4_Cl had the least significant influence. The model is deemed significant based on its *F*-value of 34.19. This kind of huge *F*-value has a 0.01% probability of being caused by noise (Table S5).

**Fig. 3:**
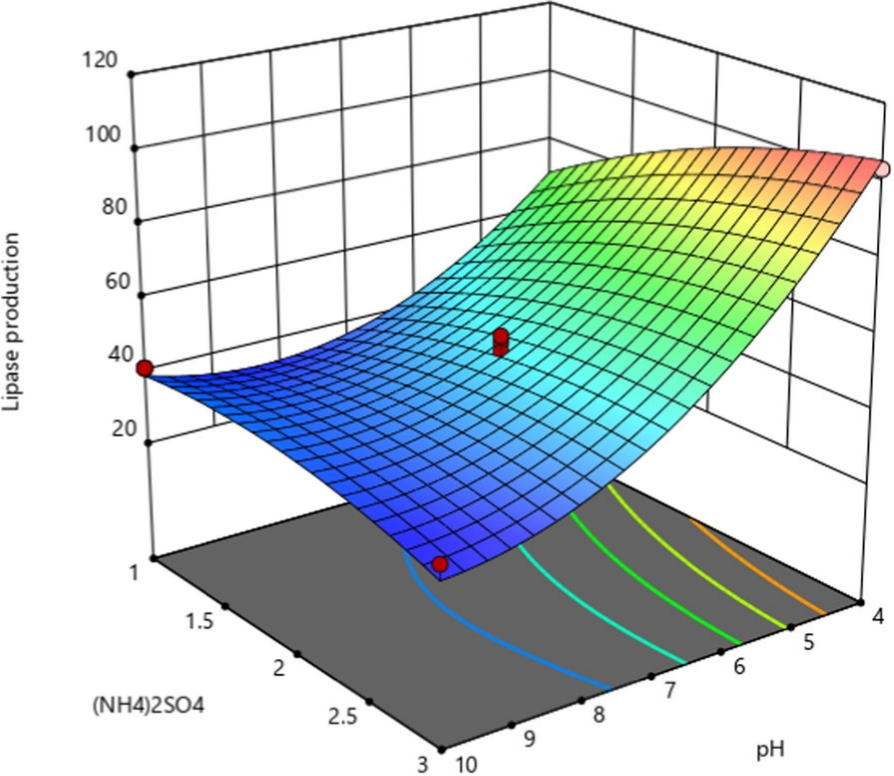
3D surface plot representing interaction effect of lipase production, pH and (NH_4_)_2_SO_4_

In order to examine the relationship between factors and determine the ideal circumstances for attaining the maximum lipase production, regression analysis was utilized to create 3D surface plots (Fig. 3). Two parameters were changed in this study while the values of the other variables were kept at their midpoints. A considerable interaction impact between pH and (NH_4_)_2_SO_4_ is revealed by the 3D plot (Fig. 3). The highest amount of lipase synthesis (103.3 U/mL) was achieved at pH 4.0 and 3 g/L of (NH_4_)_2_SO_4_ (Fig. 3).

### Validation

The goal of the validation strategy was to identify the optimal set of parameters for raising lipase production. This meant ensuring the simulation model's accuracy and refining the experimental design. An experiment was conducted using the ideal parameters recommended by the RSM optimizer in order to validate the regression model. The model and experimental data showed a significant agreement, with the measured lipase production (88.2 U/mL) closely matching the projected quantity (87.91 U/mL). As a result, the model is deemed validated (Table 2).

**Table 2:** Optimal settings of media components

Parameter settings from RSM optimizer			Y Lipase production	
pH	NH_4_Cl	(NH_4_)_2_SO_4_	Actual value	Predicted value
4.41	2.76	1.78	88.2	87.91

### Purification of lipase

At the optimum fermentation conditions of pH 4.0 and 25 °C after 3 days of fermentation using 3 g/L and 2 g/L ammonium sulphate and ammonium chloride, respectively as a nitrogen supply, the lipase was produced by the *A. terreus* AUMC 15762. The enzyme was anion-exchange chromatographed on Trilite MA 12 gel, yielding 7-pooled fractions with the highest active lipase and protein peaks. The specific activity reached the maximum (648.64 U/mg) in the most active fractions. The fractions with the greatest activity from the Trilite MA column were collected and purified further using the Sephadex G 100 column. Gel filtration of the most active lipase components recovered on Sephadex G100 yielded two significant wide peaks of lipase and protein activity (Fig. S2). After two columns processing, the specific activity of the purified lipase was increased by 17.79-fold purification recording 3571.4 U/mg with 0.07% yield (Table 3).

**Table 3:** Purification profile of lipase produced by *A. terreus* AUMC 15762

Purification step	Volume (mL)	Lipase activity (U/mL)	Total activity (U)	Protein content (mg/mL)	Total protein (mg)	Specific activity (U/mg)	Fold	Yield (%)
Production medium	1450	79.1	114,695	0.394	571.3	200.76	1	100
Ammonium sulfate	42	100	4200	0.283	11.89	353.23	1.76	2.08
Trilite MA12	14	120	1680	0.185	2.59	648.64	3.23	0.45
Sephadex G 100	7	200	1400	0.056	0.392	3571.4	17.79	0.07

### SDS-PAGE

**Fig. 4:**
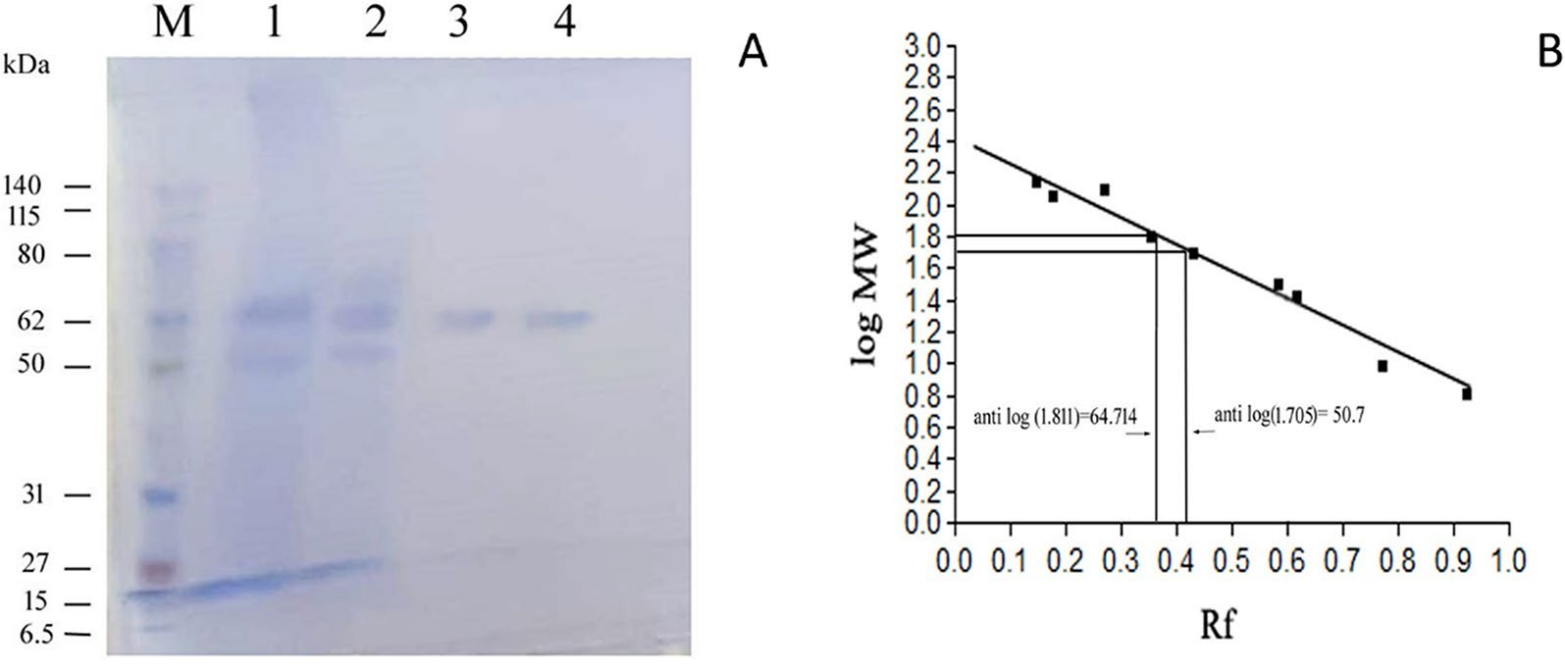
**A** SDS-PAGE illustrating the lipase purification procedures. M: Standard pre-stained marker. Lane 1: Ammonium sulfate-precipitated crude lipase. Lane 2: Purified lipase with Trilite MA-12. Lanes 3 and 4: Pure lipase isolated from Sephadex G 100. **B** Estimation of the apparent molecular weight of the pure lipase

Based on SDS-PAGE analysis, the produced lipase was fully purified into a single band with a molecular weight of 64.714 kD (Fig. 4).

### Effect of pH and temperature on the activity of pure lipase

**Fig. 5:**
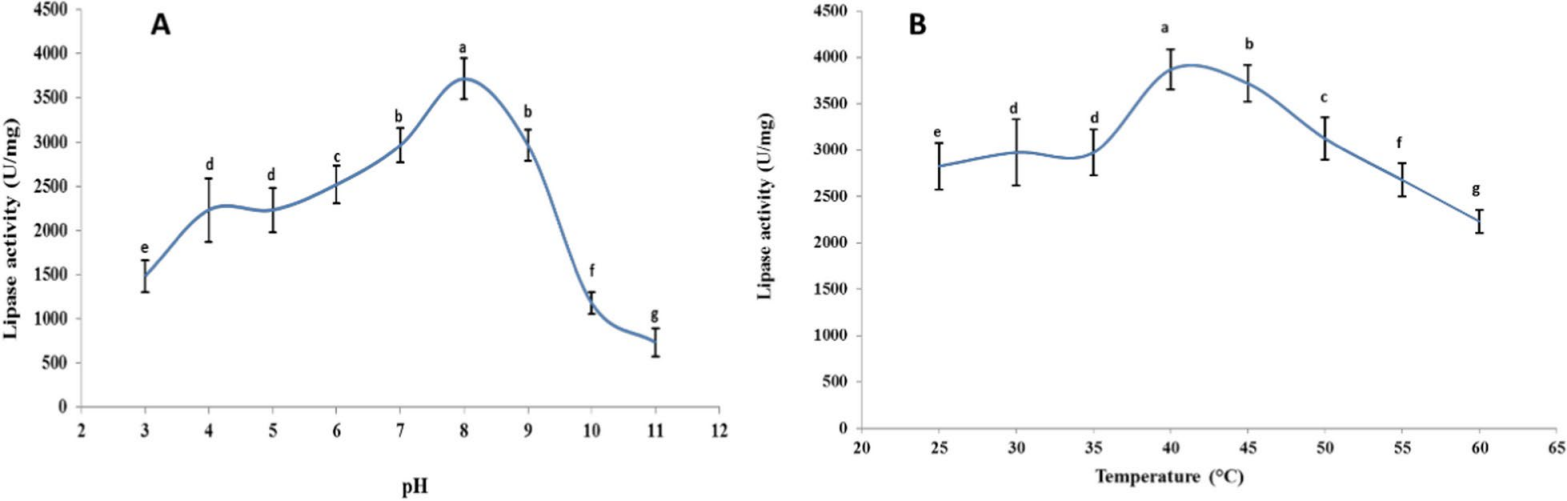
Effect of pH **A** and temperature **B** on the activity of pure lipase produced by *A. terreus* AUMC 15762 (Mean values (± SD) with different letters are significantly different (*p* > 0.05; *n* = 3)

When the pH rose to 8.0, the lipase activity increased dramatically, reaching 3714.28 ± 232.14 U/mg specific activity (Fig. 5A). At 40 °C, the lipase activity reached a peak of 3867.85 ± 214.28 U/mg (Fig. 5B).

### Substrate specificity

**Fig. 6:**
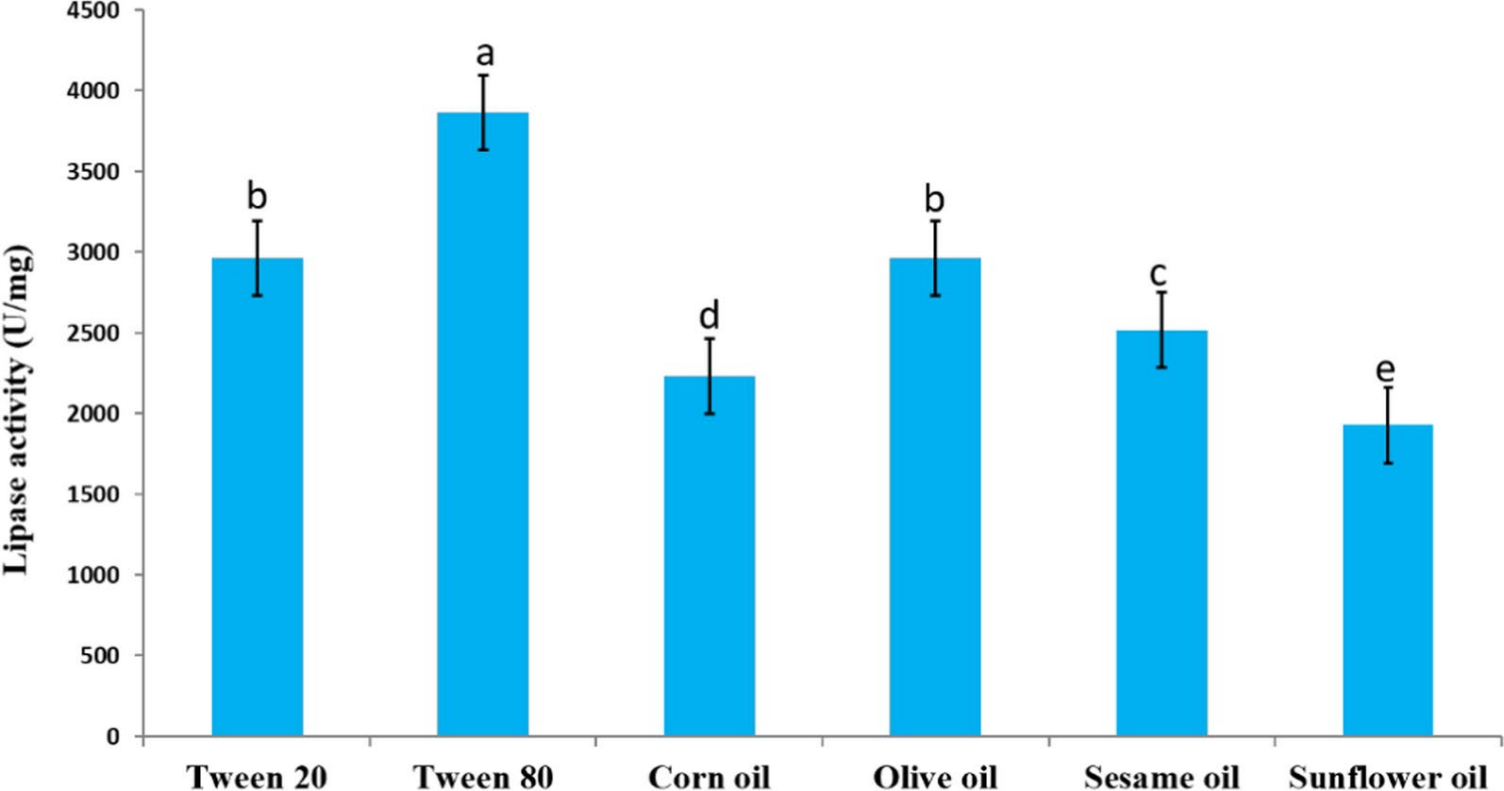
Action of the pure lipase produced by *A. terreus* AUMC 15762 on different kinds of substrates (Mean values (± SD) with different letters are significantly different (*p* ≤ 0.05; *n* = 3)

With a specific activity of 3867.85 ± 214.28 U/mg, Tween 80 was the best substrate displaying the lipase activity peak. On the other hand, 1928 ± 267.85 U/mg was the lowest specific activity recorded when using sunflower oil (Fig. 6).

### Effects of metal ions on the activity of the pure lipase

The current findings showed how certain divalent ions affected the activity of the pure lipase. When KCl and ZnSO_4_ were present, there was an increase of 115.42% in the lipase specific activity displaying specific activity of 4464.28 ± 285.71 and 4464.28 ± 196.42 U/mg, respectively. On the other hand, the lipase activity was reduced by Na^+^, Ni^2+^, Fe^2+^, Ca^2+^, Co^2+^, Mn^2+^, Mg^2+^, EDTA, and SDS (Table S6). The results of this study demonstrated how specific divalent ions impacted pure lipase activity. The specific activity of lipase was shown to rise by 115.42% in the presence of KCl and ZnSO_4_, with specific activity values of 4464.28 ± 285.71 and 4464.28 ± 196.42 U/mg, respectively. Conversely, Na^+^, Ni^2+^, Fe^2+^, Ca^2+^, Co^2+^, Mn^2+^, Mg^2+^, EDTA, and SDS all decreased the lipase activity (Table S6).

### Determination of the kinetic constants (K_m_ and V_max_)

The Lineweaver–Burk plot was used to determine the kinetic parameters. The results revealed that the computed values for K_m_ and V_max_ were 19.0 mg/mL and 1000 μmol/min, respectively (Fig. S3).

### Removal of oily spots

**Fig. 7:**
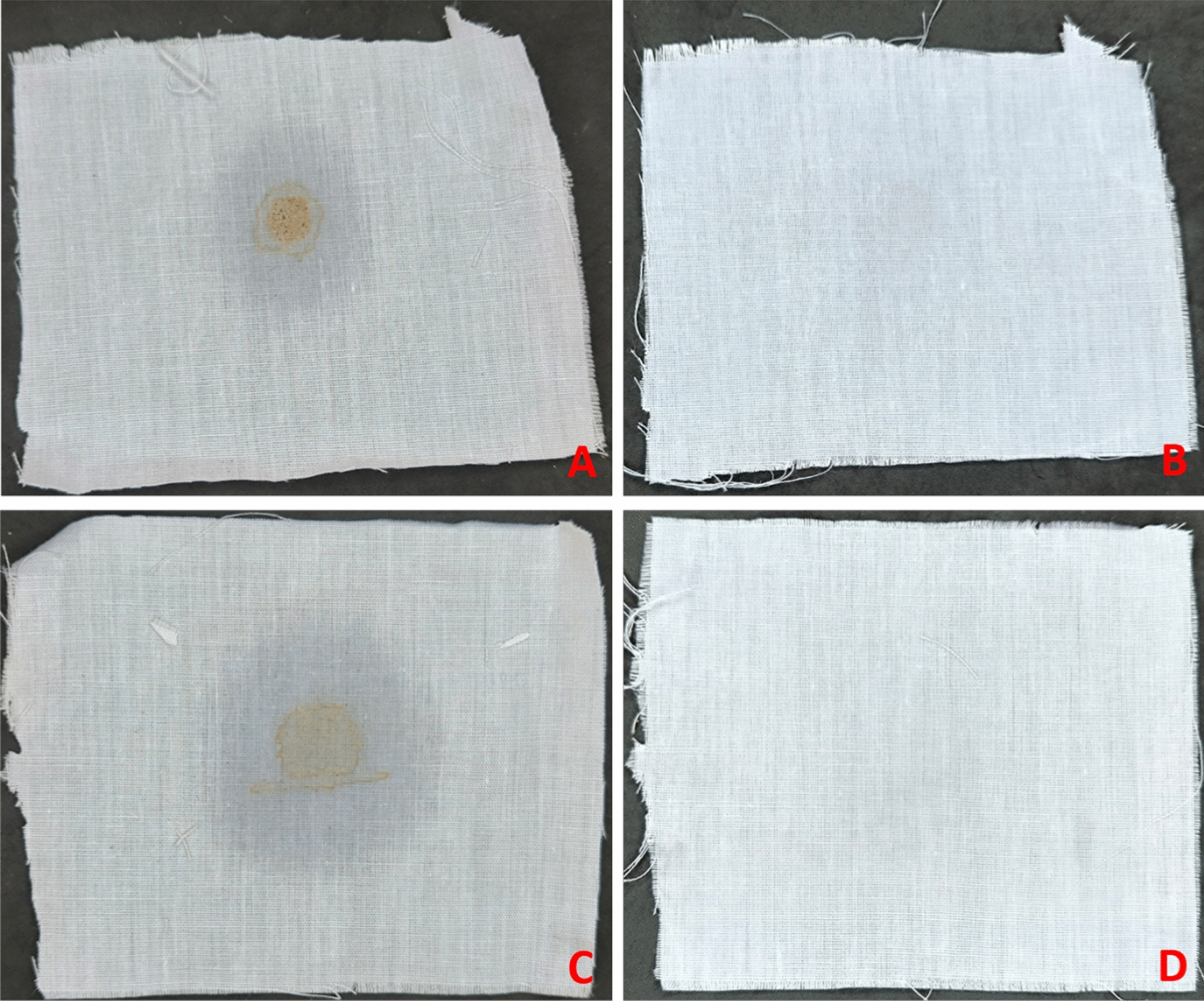
Removal of oily spots from cotton cloth pieces (10 cm × 10 cm) **A and C** Stained cotton cloth pieces with corn and olive oil **B and D** Treated cotton cloth pieces (60 min, 150 rpm at pH 8.0 and 40 °C) with the pure lipase (20 U/mL) produced by *A*. *terreus* AUMC 15762

The current study examined the potential of the pure lipase, for eliminating corn and olive oily spots. The observations revealed that, the oily spots were detached from the white cotton clothes after incubating the clothes with 20 U/mL of purified lipase for 60 min (Fig. 7). When the control (the oily stain without the addition of lipase) was compared to the treated cotton clothes, it became clear that the pure lipase, in this study, was more successful at removing oil dirt from the fiber surfaces.

## Discussion

The enzyme lipase facilitates the breakdown of triglycerides into fatty acids and glycerol, making it one of the most significant biocatalysts. Plants, bacteria, yeasts, and fungus are among the majority of species that have lipase enzymes (Ray 2015). Based on the popularity of batch fermentation and low-cost extraction techniques in modern technology, Facchini et al. (2016), concluded that fungal lipases are superior than bacterial lipases. Fungal lipases are becoming more and more used in industry due to their stability and selectivity for various substrates under a variety of chemical and physical conditions.

In this study, *Aspergillus terreus* AUMC 15762 was used to produce lipase in submerged fermentation. In the commercial and industrial world, the most important fungal species that produce lipases are related to *Acremonium*, *Alternaria*, *Aspergillus*, *Beauveria*, *Eurotium*, *Fusarium*, *Geotrichum*, *Humicola*, *Mucor*, *Ophiostoma*, *Penicillium*, *Rhizomucor*, and *Rhizopus* (Pandey et al. 2016; Joshi et al. 2019; Bharathi and Rajalakshmi 2019; Singh and Mukhopadhyay 2012). Consequently, it is essential to isolate novel fungal species with high lipase outputs in order to meet the need for lipases in the near future for both industrial and domestic uses. Further research into innovative optimization methods is required to get higher quality and larger yields of fungal lipase (Kumar et al. 2023).

The current findings corroborated those of Saleem (2008) examined the lipolytic activity of several fungal strains and found that the following fungi were lipase producers: *Aspergillus flavus*, *A. sydowii*, *A. terreus*, *Alternaria alternata*, and *Cochliobolus spicifer*. Moharram et al. (2021), discovered that most fungal strains isolated from aphids possessed the ability to produce lipolytic enzymes, with *Aspergillus niger*, *Botryotrichum atrogriseum*, *Cochliobolus spicifer*, and *Fusarium proliferatum* being the most active strains.

It could be feasible to better understand how the independent factors affect lipase production by using experimental design approaches. It was possible to determine the maximum activity levels and maximize the lipase synthesis by *A. terreus* AUMC 15762 in this work by designing experiments with PBD and RSM. After 72 h at pH 4.0 and 25 °C, the highest lipase activity (103.3 U/mL) was achieved using 3.0 g/L ammonium sulphate as the nitrogen source. In line with the current findings, the efficacy of *A. niger* C in augmenting lipase production has been assessed through the utilization of fractional factorial design (FFD) and central composite rotational design (CCRD), which aligns with the present findings. At pH 5.0, 32 °C, 4.0 g/L ammonium sulfate, and 1.0 g/L yeast extract, the maximum lipase activity of 27.46 U/mL was observed (Lima et al. 2019).

In many investigations, when response surface approach and PBD were combined, the process scale-up and commercialization of lipase production from a variety of bacteria, fungi, and yeasts became more feasible (Isiaka Adetunji and Olufolahan Olaniran 2018). In light of this, medium optimization employing RSM was utilized to enable *Pseudomonas aeruginosa* to generate lipase at an affordable price. In comparison to unoptimized medium, the results demonstrated a 5.58-fold increase in lipase synthesis (4580 U/mL) (Ruchi et al. 2008). *Aspergillus terreus* produced the highest amount of lipase (19.65 U/mL) after 96 h at pH 8.0 and 45 °C (Kaushik et al. 2010). Papagora et al. (2013) improved the lipase production from *Debaryomyces hansenii* YLL29 by using RSM. After 72 h, lipase production reached 7.44 U/mL under the appropriate optimum conditions of pH 6.4 and 30 °C due to further optimization of the selected variables using RSM. After medium optimization using statistical techniques, *Alkalibacillus salilacus* produced lipase that was overall 4.9 times higher than that of unoptimized media (Samaei-Nouroozi et al. 2015). Using PBD and RSM, the output of lipase from *Bacillus cereus* was estimated to be 343 U/mL, 1.83 times more than that of the non-optimized lipase (Vasiee et al. 2016). The activity of lipase from *A. niger* HN1 strain in optimized medium was 40% higher (147.65 U mL/mL) than in un-optimized medium (105.19 U/mL), while it was 38% higher for *A. niger* LPF-5 strain in optimized medium (Sharma et al. 2016). Under ideal circumstances, *Bacillus aryabhattai* SE3-PB lipase production peaked (264.02 U/mL) at pH 7.6 and 40 °C after 72 h, according to the study using face-centered central composite design (FCCCD) (Isiaka Adetunji and Olufolahan Olaniran 2018). Plackett–Burman and Central Composite Designs (CCD) were employed to investigate various aspects of *Aspergillus fumigatus*'s lipase production, which has been shown to reach its maximal level (6.22 U/mL) at pH 10.0 and 45 °C after 72 h (Mehta et al. 2019). Lipase production by *Serratia marcescens* VT 1 reached the maximum (55.95 U/mL) using PBD and RSM Krishnankutty (2024).

The lipase in this investigation was purified 17.79 times through the application of ammonium sulphate precipitation and column chromatography (Trilite MA 12 and Sephadex G 100). Considering this, ammonium sulfate precipitation is the most often utilized technique for precipitating proteins (Yagmurov et al. 2017). Ammonium sulfate is a cost-effective salt with excellent water solubility and chemotropic properties that increase water entropy. It thus reduces the flexibility of proteins and enhances hydrophobic interactions. Consequently, the third structure of the protein hardens and prevents the domains from breaking (Coligan 2001). According to Tripathi et al. (2014), ammonium sulphate precipitation and Sephadex G–100 gel column chromatography were used in sequence to purify the lipase from *Geobacillus stearothermophilus* AH22. Niyonzima and More (2015) isolated lipase from *Microbacterium* sp. using a variety of methods, such as ammonium sulfate precipitation and Sephadex G–75 gel column chromatography. The maximum amount of hydrolytic activity was shown by the purified lipase. Similar purification of lipase from *Bacillus methylotrophicus* PS3 was achieved by means of ammonium sulfate and Sephadex G–100 column chromatography (Sharma et al. 2017).

The results of this study showed that pH 8.0 and 40 °C were the ideal values for the pure lipase of *A. terreus* AUMC 15762, attaining 3714.28 ± 232.14 U/mg and 3867.85 ± 214.28 U/mg specific activity, respectively. In agreement to the current findings, pH 8.0 and 60 °C were ideal for exhibiting the lipase of *Humicola lanuginosa* S–38 at its maximal specific activity of 1750 U/mg (Liu et al. 1973). The lipase produced by *Alkalibacillus salilacus* was optimally active at pH 8.0 and 40 °C (Samaei-Nouroozi et al. 2015). The lipase produced by *A. niger* exhibited its maximum enzymatic activity at pH 7.5 and 40 °C (Alabdalall et al. 2020).

SDS–PAGE results in this study revealed that the purified lipase’s molecular weight was 64.714 kDa. In light of this finding, numerous reports of lipases with variable molecular weights originating from different microorganisms have been published. For instance, the lipase from *Pichia lynferdii* Y-7723 was isolated by Bae et al. (2014) using chromatographic techniques, and the isolated enzyme had a molecular weight of 40 kDa on SDS-PAGE. The molecular weights of the *Rhizomucor miehei* NRRL 5282 and *Rhizopus oryzae* NRRL 1526 lipases were estimated to be between 55 and 35 kDa, respectively (Takó et al. 2017). It has been found that most lipases found in the *Penicillium* genus have molecular masses between 25 and 43 kDa (Li and Zong 2010; Bian et al. 2005; Ruiz et al. 2001). The molecular mass of *Penicillium expansum* strain PED–03 (Lianghua et al. 2007) and *Penicillium crustosum* strain P22 (Hasnaoui et al. 2022) was 28 kDa for both enzymes.

The present results showed that the presence of K^+^ and Zn^2+^ ions slightly increased the specific activities of the enzyme, which were 4464.28 ± 285.71 and 4464.28 ± 196.42 U/mg, respectively, compared to the control value (3867.85 ± 214.28 U/mg). On the other hand, Na^+^, Ni^2+^, Cu^2+^, Fe^2+^, Ca^2+^, Co^2+^, Mn^2+^, Mg^2+^, EDTA, and SDS, decreased the lipase activity. Metallic ions are common agents needed by enzymes in several metabolic processes (Fu and Xi 2020). According to Mahfoudhi et al. (Mahfoudhi et al. 2022), metal ions play a significant role in the structure and operation of many different enzymes, including lipases.

Ions can interact (or chelate) with proteins to form complexes that can affect protein stability when they are used as an enzyme’s co-substrate, substrate, or co-factor (Bauduin et al. 2004). It was thought that an ion's ability to change the structure of water—also referred to as a “physical” effect—was primarily responsible for its ion selectivity (Bauduin et al. 2004; Boström et al. 2004). Strongly hydrated water ions that support an object's structure are known as kosmotropes, whereas weakly hydrated water ions that undermine an object’s structure are known as chaotropes, or “structure-breakers.” As opposed to chaotropes, which are frequently enormous and negatively charged, kosmotropes are usually small and positively charged. In actuality, kosmotropic and highly hydrated multivalent ions exist in all of them (Krestov 1991; Zhao 2005; Das et al. 2016).

According to Sztajer et al. (Sztajer et al. 1992), the influence of the interfacial area between the substrate and the enzyme may be the cause of the reduction in lipase activity caused by EDTA. Toida et al. (1995) found suppression of activity of *Aspergillus oryzae*’s lipase by Cu^2+^, Fe^3+^, Hg^2+^, Zn^2+^ and Ag^+^. According to Tiwari et al. (2011), the presence of Mg^2+^, Ba^2+^, and Ca^2+^ boosted lipase activity, whereas the presence of Ag^+^ inhibited it. The ions Al^3+^, Ca^2+^, Mn^2+^, Zn^2+^, and Hg^2+^ were found to enhance the extracellular lipase activity of *Candida kikuchii* (Costa-Silva et al. 2014). Yang et al. (2016) claimed that the enzymatic activity of MgSO_4_ was hindered. Sahoo et al. (2017) similarly accomplished the catalytic result of the enzyme in the presence of the identical ions. *Aspergillus japonicus* lipase was stable in the presence of Ca^2+^ but was inhibited by Mn^2+^ and Hg^2+^ (Jayaprakash and Ebenezer 2012; Kumar et al. 2023). *Candida rugosa* has shown increased lipase activity when exposed to Ca^2+^ ions (Kumar et al. 2023b; Katiyar and Ali 2013). Saranya and Ramachandra (2020) discovered that Ca^2+^, Mg^2+^, Na^+^, and K^+^ have no effect on the activity of lipase produced from *Cladosporium tenuissimum*. The lipase activity isolated from *Aspergillus oryzae* was also found to be inhibited by Cu^2+^, Fe^3+^, Hg^2+^, Zn^2+^, and Ag^+^ (Toida et al. 1995; Kumar et al. 2023).

Using Tween 80 as the substrate in this study produced a specific activity of 3867.85 ± 214.28 U/mg. There was a suggested explanation for these contradictory elements. The structure of the enzyme was altered when water bonded to it. The detergent may benefit from these alterations, which increase the enzyme's enzymatic activity and effective substrate accessibility (Joseph et al. 2008). Because these surfactants disrupt the crucial structural links that underpin enzymatic activity, it is possible that they inhibit the enzymes (Yao et al. 2013).

The K_m_ and V_max_ in this study were calculated as 19.0 mg/mL and 1000 μmol/min, respectively. The lower K_m_ value indicates high substrate-enzyme affinity, while a larger V_max_ value guarantees high lipase catalytic efficiency (Sharma et al. 2001). The K_m_ and V_max_ values for *Rhizopus* sp. lipase, as reported by Koblitz and Pastore (2006), were 2.4 mg/mL and 277.8 μmol/min, respectively. Lai et al. (1999) state that the equilibrium hypothesis (Michaelis–Menten equation) indicates that the rate is comparable to that. This makes it possible to plot the relationship between the starting rate and the concentration of the other substrate.

This study looked into the viability of removing different greasy areas with *A. terreus*'s lipase. The findings revealed that the oily patches on the white cotton garment totally detached from the apparel after a 60-min incubation time. In this study, it was discovered that lipase was more successful in removing oil stains from the fiber surfaces at pH 8.0 and 40 °C. Today’s heavy-duty powder and automatic dishwashing detergents use a number of lipases to increase their detergency and prevent scaling (Bora and Bora 2012). Similarly, compared to the chemicals in conventional detergents, these enzymes are less harmful and leave no dangerous residues (Bharathi and Rajalakshmi 2019). In fact, a number of studies have shown that thermostable alkaline lipases produced from *Pseudomonas aeruginosa* (Grbavčić et al. 2011) and *Bacillus sonorensis* (Nerurkar et al. 2013) were effective when paired with detergent in removing oily stains from cotton fabrics. Additionally, the lipase from *B. subtilis* showed good washing performance at 30 °C and an alkaline pH (pH 8.0–11.0) (Eggert et al. 2002).

## Supplementary Information

Supplementary Material 1. Table S1: Screening of independent parameters and their levels used in PBD; Table S2: The influencing factors and their levels in Box–Behnken design BBD; Table S3: Preliminary and secondary screening of the lipolytic activity by 141 fungal isolates from 15 Desert soil samples collected from Sohag, Qena, and Aswan Governorates during February 2019; Table S4: ANOVA for selected factorial model; Table S5: ANOVA test results for lipase production; Table S6: Effects of metal ions on the activity of the pure lipase produced by A. terreus AUMC 15762 (Mean values (±SD) with different letters are significantly different (p ≤ 0.05; n = 3); Fig. S1: Fig. S1 Aspergillus terreus AUMC 15762. (A), Seven-day-old colonies on CYA at 25 °C (B), conidiophores and conidial heads. (C), smooth, globose conidia (Scale bars = 20 µm). Fig. S2: Purification of lipase in crude extract by anion exchange resin Flow rate = 15 ml/h, Fraction volume = 3 ml; Eluent = 100 mM phosphate buffer (pH 8.0); Fig. S3: Line weaver–Burk plot for the pure lipase produced by A. terreus AUMC 15762.

## Data Availability

All data related to this manuscript is incorporated in the manuscript and the supplementary material.
