# Supplementary material for: Production of alkaline lipase by Aspergillus terreus AUMC 15762 for laundry application
Source: AMB Express. 2025 Apr 13;15:64. doi: 10.1186/s13568-025-01865-x (PMC11994561; doi:10.1186/s13568-025-01865-x)
Supplement: Supplementary file 1 — Supplementary Material 1. Table S1: Screening of independent parameters and their levels used in PBD; Table S2: The influencing factors and their levels in Box–Behnken design BBD; Table S3: Preliminary and secondary screening of the lipolytic activity by 141 fungal isolates from 15 Desert soil samples collected from Sohag, Qena, and Aswan Governorates during February 2019; Table S4: ANOVA for selected factorial model; Table S5: ANOVA test results for lipase production; Table S6: Effects of metal ions on the activity of the pure lipase produced by A. terreus AUMC 15762 (Mean values (±SD) with different letters are significantly different (p ≤ 0.05; n = 3); Fig. S1: Fig. S1 Aspergillus terreus AUMC 15762. (A), Seven-day-old colonies on CYA at 25 °C (B), conidiophores and conidial heads. (C), smooth, globose conidia (Scale bars = 20 µm). Fig. S2: Purification of lipase in crude extract by anion exchange resin Flow rate = 15 ml/h, Fraction volume = 3 ml; Eluent = 100 mM phosphate buffer (pH 8.0); Fig. S3: Line weaver–Burk plot for the pure lipase produced by A. terreus AUMC 15762. [file 13568_2025_1865_MOESM1_ESM.docx]

**Production of Alkaline Lipase by *Aspergillus terreus* AUMC 15762 for Laundry Application**

Osama Abdel-Hafeez Mohamed Al-Bedak^1,2,^*, Ahmed Mohamed Ahmed Ali Ramadan^3^, Hussein H. EL–Sheikh^3^, Reda M. Shehata^3^

^1^Assiut University Mycological Centre, Assiut 71511, Egypt; [osamaalbedak@science.au.edu.eg](mailto:osamaalbedak@science.au.edu.eg) (O. A. M. A.)

^2^ERU Science & Innovation Center of Excellence, Egyptian Russian University, Badr city 11829, Egypt

^3^Department of Botany & Microbiology, Faculty of Science, Al Azhar University, Cairo, 11511, Egypt;
[T374891@azharonline.edu.eg](mailto:T374891@azharonline.edu.eg) (A.M.A.A.R.); [husseinhosny.221@azhar.edu.eg](mailto:husseinhosny.221@azhar.edu.eg) (H.H.E.–S.) [redamostafa.18@azhar.edu.eg](mailto:redamostafa.18@azhar.edu.eg) (R.M.S.)

^*^Corresponding author: [osamaalbedak@science.au.edu.eg](mailto:osamaalbedak@science.au.edu.eg)

**Table S1.** Screening of independent parameters and their levels used in PBD.

| **No** | **Factors** | **Symbol** | **Low level (–1)** | **High level (+1)** |
| --- | --- | --- | --- | --- |
| 1 | Temperature | A | 25 | 40 |
| 2 | pH | B | 4 | 9 |
| 3 | Tween 80 | C | 0.5 | 1.5 |
| 4 | Peptone | D | 1 | 3 |
| 5 | Yeast extract | E | 1 | 3 |
| 6 | Sodium nitrate | F | 1 | 3 |
| 7 | Ammonium chloride | G | 1 | 3 |
| 8 | Urea | H | 1 | 3 |
| 9 | Ammonium sulphate | J | 1 | 3 |

**Table S2.** The influencing factors and their levels in Box–Behnken design BBD.

| **Independent variable** | **Symbol** | **Actual value of code** | | |
| --- | --- | --- | --- | --- |
|  |  | –1 | 0 | +1 |
| pH | X_1_ = A | 4 | 7 | 10 |
| Ammonium chloride NH_4_Cl | X_2_ = B | 1 | 2 | 3 |
| Ammonium sulphate (NH_4_)_2_SO_4_ | X_3_ = C | 1 | 2 | 3 |

**Table S3**. Preliminary and secondary screening of the lipolytic activity by 141 fungal isolates from 15 Desert soil samples collected from Sohag, Qena, and Aswan Governorates during February 2019.

| **Taxa** | **No. of isolates** | **Positive isolates** | **Preliminary screening (mm)** | | | **Secondary screening (U/mL)** | | |
| --- | --- | --- | --- | --- | --- | --- | --- | --- |
|  |  |  | H | M | L | H | M | L |
| ***Acremonium*** *roseolum* | **1** | 1 | 1 | – | – | – | – | 1 |
| *Acrophialophora fusispora* | **3** | 0 | – | – | – | – | – | – |
| *Alternaria alternata* | **2** | **0** | – | – | – | – | – | – |
| ***Aspergillus*** *aureoterreus* | 8 | 5 | 3 | 2 | – | – | 2 | 1 |
| *A. flavipes* | 1 | 1 | – | – | 1 | – | – | – |
| *A. flavus* | 13 | 10 | 3 | 2 | 5 | – | 2 | 1 |
| *A. nidulans* | 11 | 5 | – | 4 | 1 | – | – | – |
| *A. niger* | 12 | 10 | 8 | 2 | – | 1 | 7 | – |
| *A. oryzae* | 8 | 2 | 1 | 1 | – | – | 1 | – |
| *A. parasiticus* | 2 | 1 | – | – | 1 | – | – | – |
| *A. pseudodeflectus* | 5 | 2 | 2 | – | – | 1 | 1 | – |
| *A. tamarii* | 2 | 2 | 2 | – | – |  | 2 | – |
| *A. terreus* | 13 | 13 | 10 | 3 | – | 3 | 5 | 2 |
| *A. tubingensis* | 2 | 2 | 2 |  | – | 1 | 1 | – |
| *A. ustus* | 6 | 2 | 1 | 1 | – | – | 1 | – |
| *A. versicolor* | 1 | 1 | 1 | – | – | – | – | 1 |
| ***Bipolaris*** *australiensis* | 2 | 2 | 1 | – | 1 | – | – | 1 |
| *B. papendorfii* | 2 | 2 | 1 | – | 1 | – | – | 1 |
| ***Chaetomium*** *globosum* | 2 | 0 | – | – | – | – | – |  |
| ***Chrysosporium*** *tropicum* | **2** | 0 | – | – | – | – | – | – |
| ***Cdeophoma*** *ericola* | **1** | 0 | – | – | – | – | – | – |
| ***Cladosporium*** *cladosporioides* | **1** | 0 | – | – | – | – | – | – |
| *C. oxysporum* | 1 | 0 | – | – | – | – | – | – |
| ***Corynascus*** *sepedonium* | **1** | 0 | – | – | – | – | – | – |
| ***Curvularia*** *spicifera* | **1** | 0 | – | – | – | – | – | – |
| ***Epicoccum*** *nigrum* | **2** | **0** | – | – | – | – | – | – |
| ***Fusarium*** *oxysporum* | 6 | 1 | – | – | 1 | – | – | – |
| *F. solani* | 3 | 4 | – | 2 | 2 | – | – | – |
| ***Graphium*** *penicillioides* | **4** | 0 | – | – | – | – | – | – |
| ***Humicola*** *grisea* | **1** | 0 | – | – | – | – | – | – |
| ***Monodictys* s**p**.** | **2** | **0** | – | – | – | – | – | – |
| ***Penicillium*** *chrysogenum* | 2 | 3 | 1 | 2 | – | 1 | – | – |
| *P. crustosum* | 2 | 3 | 2 | 1 |  | 1 | 1 | – |
| *P. verruculosum* | 4 | 2 | 1 | 1 | – | – | 1 | – |
| ***Pestalotia* s**p***.*** | **1** | 0 | – | – | – | – | – | – |
| ***Phoma* s**p**.** | **1** | 0 | – | – | – | – | – | – |
| ***Purpureocillium*** *lilacinum* | **3** | 0 | – | – | – | – | – | – |
| ***Trichoderma*** *harzianum* | **7** | 0 | – | – | – | – | – | – |
| **Total** | **141** | **74** | **40** | **21** | **13** | **8** | **24** | **8** |
| **No. of genera** | **21** | **5** | **4** | **3** | **3** | **2** | **2** | **3** |
| **No. of species** | **38** | **22** | **17** | **12** | **8** | **6** | **11** | **7** |

**Table S4.** ANOVA for selected factorial model.

| **No** | **Code** | **Source** | **Sum of Squares** | **Df** | **Mean square** | **F value** | ***p* value** |
| --- | --- | --- | --- | --- | --- | --- | --- |
|  |  | Model | 1356.12 | 8 | 169.51 | 44.54 | 0.0049 |
| 1 | A | Temperature | 1.13 | 1 | 1.13 | 0.2957 | 0.6244 |
| 2 | B | pH | 178.06 | 1 | 178.06 | 46.79 | 0.0064 |
| 3 | C | Tween 80 | 6.08 | 1 | 6.08 | 1.6 | 0.2954 |
| 4 | D | Peptone | 11.91 | 1 | 11.91 | 3.13 | 0.175 |
| 5 | F | Sodium nitrate | 15.68 | 1 | 15.68 | 4.12 | 0.1354 |
| 6 | G | NH_4_Cl | 885.89 | 1 | 885.89 | 232.76 | 0.0006 |
| 7 | H | Urea | 10.26 | 1 | 10.26 | 2.7 | 0.1992 |
| 8 | J | (NH_4_)_2_SO_4_ | 247.11 | 1 | 247.11 | 64.93 | 0.004 |
|  |  | Residual | 11.42 | 3 | 3.81 |  |  |
|  |  | Cor Total | 1367.53 | 11 |  |  |  |
|  |  | Model | 1356.12 | 8 | 169.51 | 44.54 | 0.0049 |

*p* ≤0.05 was considered to be significant R^2^ = 0.9917, R^2^ (adj) = 0.9694, R^2^ (pred) = 0.8664

**Table S5.** ANOVA test results for lipase production

| **No** | **Code** | **Source** | **Sum of Squares** | **Df** | **Mean square** | **F value** | ***p* value** |
| --- | --- | --- | --- | --- | --- | --- | --- |
|  |  | Model | 8230.1 | 7 | 1175.7 | 34.2 | < 0.0001 |
| 1 | A | pH | 5779.9 | 1 | 5779.9 | 168.1 | < 0.0001 |
| 2 | B | NH_4_Cl | 195.0 | 1 | 195.0 | 5.7 | 0.0411 |
| 3 | C | (NH_4_)_2_SO_4_ | 326.8 | 1 | 326.8 | 9.5 | 0.0131 |
| 4 | AC | pH*(NH_4_)_2_SO_4_ | 481.8 | 1 | 481.8 | 14.0 | 0.0046 |
| 5 | A^2^ | pH² | 1247.8 | 1 | 1247.8 | 36.3 | 0.0002 |
| 6 | B² | NH_4_Cl² | 93.6 | 1 | 93.6 | 2.7 | 0.1334 |
| 7 | C² | (NH_4_)_2_SO_4_² | 111.4 | 1 | 111.4 | 3.2 | 0.1054 |
|  |  | Residual | 309.5 | 9 | 34.4 |  |  |
|  |  | Lack of Fit | 184.5 | 5 | 36.9 | 1.2 | 0.4483 |
|  |  | Pure Error | 124.9 | 4 | 31.2 |  |  |

*p* <0.05 were considered to be significant R^2^ = 0.96, R^2^ (adj) = 0.93, R^2^ (pred) = 0.83.

**Table S6.** Effects of metal ions on the activity of the pure lipase produced by *A. terreus* AUMC 15762 (Mean values (±SD) with different letters are significantly different (*p* ≤ 0.05; *n* = 3).

| **Metal ions and inhibitors** | **Specific activity (U/mg)** | **Residual activity (%)** |
| --- | --- | --- |
| Control | 3867.85±214.28^b^ | 100±5.5 |
| Na | 3714.2±285.7^c^ | 96.02±7.3 |
| K | 4464.3±321.4^a^ | 115.42±8.3 |
| Ca | 3125±303.5^g^ | 80.79±7.8 |
| Mg | 3267.8±250^f^ | 84.48±6.4 |
| Zn | 4464.3±214.2^a^ | 115.42±5.5 |
| Mn | 3410.7±232.1^e^ | 88.18±6.0 |
| Fe | 2964.2±303.5^i^ | 76.63±7.8 |
| Cu | 3571.4±303.5^d^ | 92.33±7.8 |
| Ni | 3714.2±196.4^c^ | 96.02±5.0 |
| Co | 2821.4±142.8^j^ | 72.94±3.6 |
| SDS | 2821.4±303.5^j^ | 72.94±7.8 |
| EDTA | 2964.2±267.8^h^ | 76.63±6.9 |


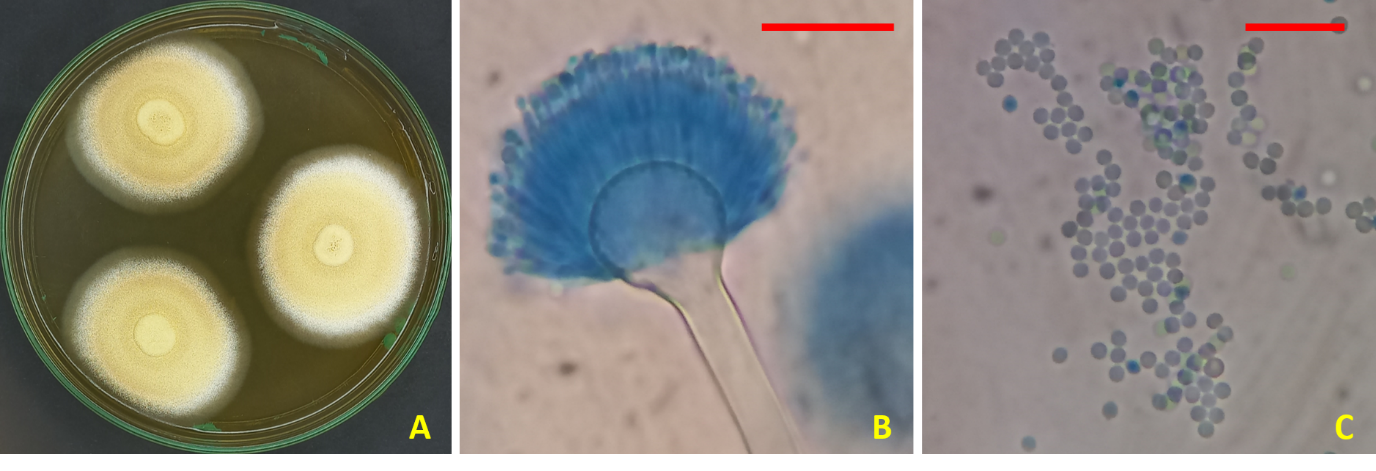


**Fig. S1** *Aspergillus terreus* AUMC 15762. **(A),** Seven-day-old colonies on CYA at 25 °C **(B),** conidiophores and conidial heads. **(C),** smooth, globose conidia (Scale bars = 20 µm).

**
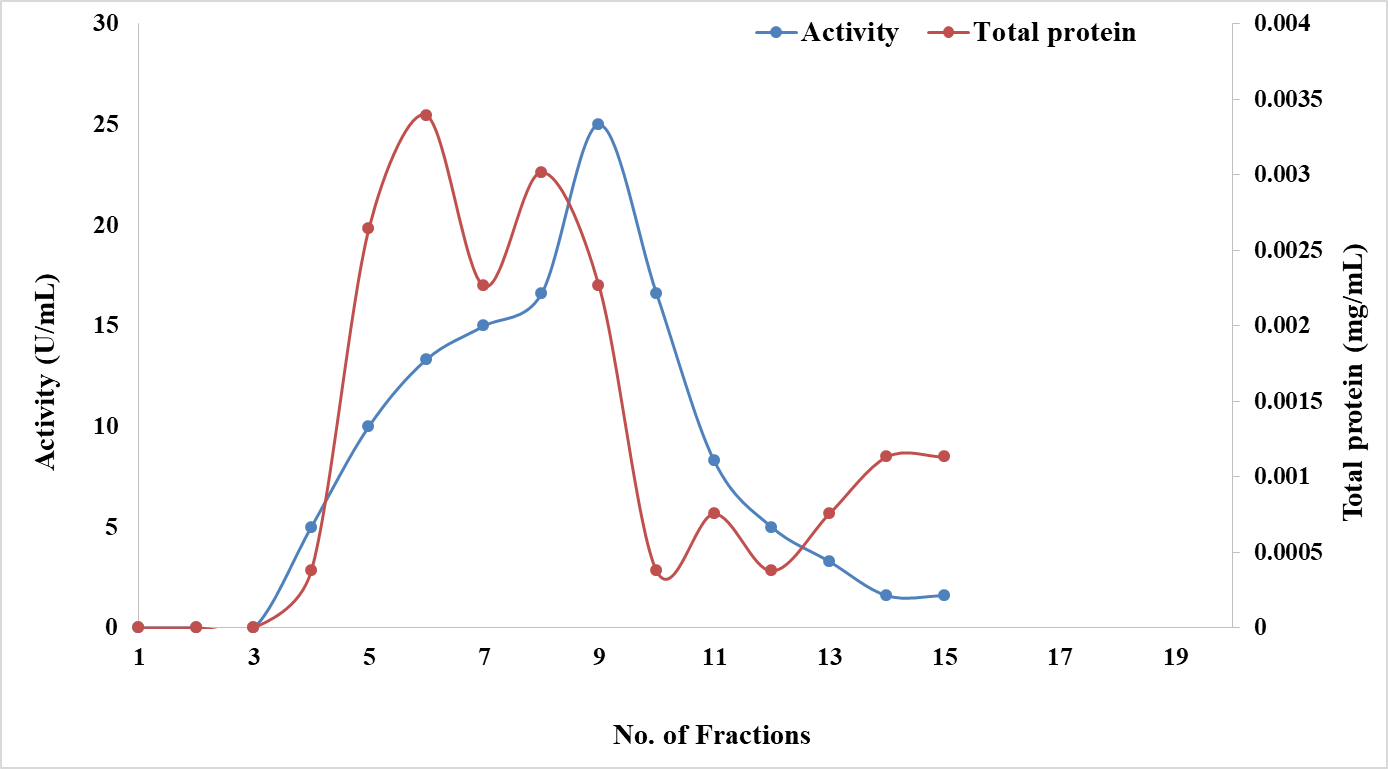
**

**Fig. S2** Purification of lipase by Trilite MA12 anion exchange resin


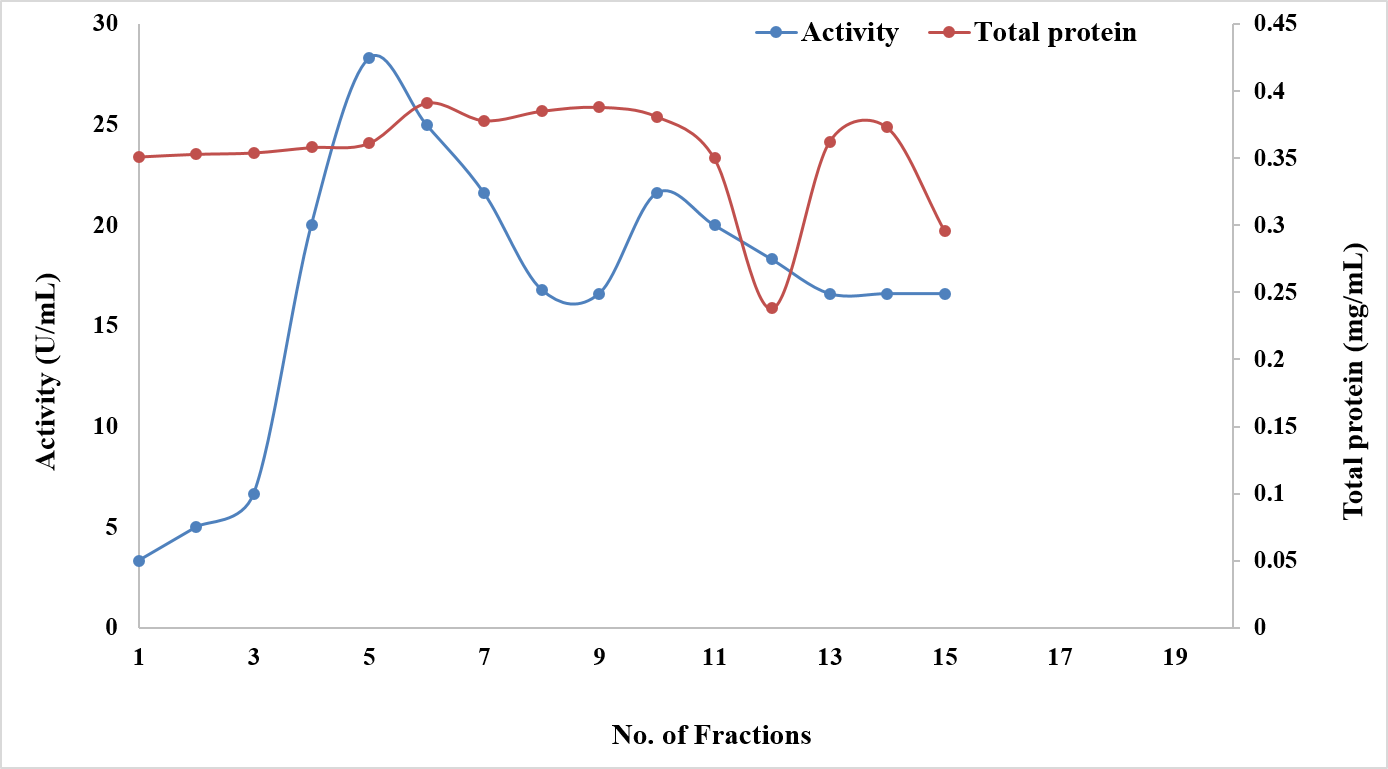


**Fig. S3** Purification of lipase by Sephadex G 100

**
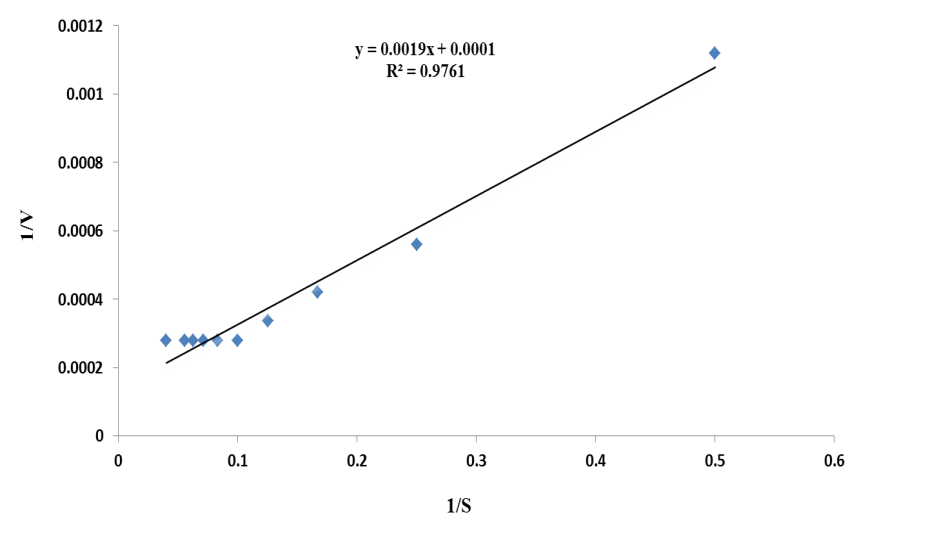
**

**Fig. S4** Line weaver–Burk plot for the pure lipase produced by *A. terreus* AUMC 15762.
